# The impact of surgically induced ischaemia on protein levels in patients undergoing rectal cancer surgery

**DOI:** 10.1038/sj.bjc.6603362

**Published:** 2006-10-03

**Authors:** G K Atkin, F M Daley, S Bourne, R Glynne-Jones, J M A Northover, G D Wilson

**Affiliations:** 1 Gray Cancer Institute, Mount Vernon Hospital, Northwood, Middlesex HA6 2RN, UK; 2 Department of Radiotherpy, Mount Vernon Hospital, Northwood, Middlesex HA6 2RN, UK; 3 Colorectal Cancer Unit, St Mark's Hospital, Harrow HA1 3UJ, UK; 4 Karmanos Cancer Institute, Wayne State University, Detroit, MI 48201-2013, USA

**Keywords:** colorectal cancer, gene expression, thymidylate synthase, hypoxia

## Abstract

The goal of targeted therapy has driven a search for markers of prognosis and response to adjuvant therapy. The surgical resection of a solid tumour induces tissue ischaemia and acidosis, both potent mediators of gene expression. This study investigated the impact of colorectal cancer (CRC) surgery on prognostic and predictive marker levels. Tumour expression of thymidylate synthase, thymidine phosphorylase, cyclin A, vascular endothelial growth factor (VEGF), carbonic anhydrase-9, hypoxia inducible factor-1*α*, and glucose transporter-1 (GLUT-1) proteins was determined before and after rectal cancer surgery. Spectral imaging of tissue sections stained by immunohistochemistry provided quantitative data. Surgery altered thymidylate synthase protein expression (*P*=0.02), and this correlated with the change in the proliferation marker cyclin A. The expression of hypoxia inducible factor-1*α*, VEGF, and GLUT-1 proteins was also different following surgery. Colorectal cancer surgery significantly impacts on intratumoral gene expression, suggesting archival specimens may not accurately reflect *in situ* marker levels. Although rectal cancer was the studied model, the results may be applicable to any solid tumour undergoing extirpation in which molecular markers have been proposed to guide patient therapy.

There has been considerable recent interest in markers of tumour prognosis and response to adjuvant therapy in a range of tumour types, including colorectal cancer (CRC). It is hoped these markers will allow targeted tumour therapy while minimising the toxicity of inefficacious agents. Thymidylate synthase (TS) is a key enzyme in DNA synthesis and is the main site of action of the chemotherapeutic agent 5-fluorouracil (5-FU) (van der Wilt and Peters, 1994). It is the most widely studied prognostic and predictive marker in CRC, with low TS levels predicting a better outcome to 5-FU based chemotherapy (Aschele *et al*, 2002), and high TS expression being associated with a poor prognosis (Allegra *et al*, 2003). However, previous reports of TS display methodological heterogeneity, in that TS expression within preoperative biopsies (Okonkwo *et al*, 2001) as well as postoperative archival tumour sections (Edler *et al*, 2000), has been correlated with treatment outcome.

The resection of a solid tumour, such as a CRC, often involves early clamping of the vascular pedicle to facilitate the surgical dissection and aid tumour extirpation. Interrupting the vascular inflow leads to tumour hypoxia and acidosis (Parkins *et al*, 1997), both of which are potent mediators of gene expression (Helfman and Falanga, 1993). In addition, delays in tissue fixation following extirpation reduce the efficacy of subsequent protein and mRNA analysis (Almeida *et al*, 2004).

We have previously shown in an experimental CRC model that tumour vascular clamping significantly alters gene expression levels (Atkin *et al*, 2006). There are no formal clinical guidelines regarding the rapidity of tumour fixation following extirpation, and if CRC surgery and tissue processing methods do alter gene expression levels, it may be that marker expression in postoperative tumour samples does not reflect true *in situ* levels. Therefore, the aim of this study was to determine the effect of surgery on marker expression in patients with CRC, looking in particular at changes in thymidylate synthase. The relationships between TS expression and the levels of cyclin A, a cell proliferation marker, and the hypoxia-related protein hypoxia inducible factor-1*α* (HIF-1*α*) are also presented.

## MATERIALS AND METHODS

### Study patients

Patients undergoing surgical resection of histologically proven rectal cancer were recruited over a 2-year period. There were no exclusion criteria. Standard curative resections and anaesthetic techniques were performed in all cases. Local ethics committee approval was obtained and all patients gave informed consent.

Five preoperative tumour samples were obtained on the day of surgery, immediately prior to the surgical resection. A further five biopsies were obtained immediately following tumour extirpation. In addition, for each patient a control sample of rectal mucosa was obtained before and after surgery, at the same time as tumour biopsies. Tumour and mucosal sampling was confirmed by examination of haematoxylin and eosin stained sections by an independent, experienced histopathologist.

Each tumour and control mucosal biopsy was stored in 10% neutral buffered formalin solution (Sigma, Poole, UK) for subsequent protein analysis by immunohistochemistry. For all patients, the duration of tumour ischaemia (defined as time of arterial pedicle clamping until postoperative biopsy) was noted, as well as the time between extirpation and postoperative tumour sampling. In all cases, the true clinical duration of tumour ischaemia was longer than that noted in this study, as the postoperative biopsies were obtained before actual fixation of the surgical specimen by the operating theatre staff.

### Immunohistochemistry

Table 1 shows the proteins and the conditions used for immunohistochemistry. Sections (4 *μ*m) were dewaxed in xylene for 5 min and rehydrated through graded alcohol (100, 90, and 70%) to water. Heat mediated antigen retrieval was performed using 250 ml 10 mM citric acid pH 6 for all markers, apart from vascular endothelial growth factor (VEGF) (0.1 M Tris-HCL pH 10) and CA-9 (no pretreatment), by boiling the sections in an 800 W microwave oven (Panasonic NN-6453BBPQ, 2450 MHz).

For all markers apart from CA-9 and HIF-1*α*, sections were transferred to the DAKO Autostaining machine (DAKO, UK) containing peroxidase block (DAKO, S2023), the detection reagents (ChemMate HRP, DAKO K5001), and anti-human primary antibody diluted in antibody diluent. The Autostainer programme included 5 min in peroxidase block, 1 h incubation in primary antibody, 30 min incubation in ChemMate secondary and tertiary reagents and 5 min in diaminobenzidine (DAB) substrate.

Sections were stained for HIF-1*α* using the DakoCytomation CSA II signal amplification system (DAKO Corporation, Carpinteria, USA). In summary, the sections were first incubated with 3% hydrogen peroxide for 5 min to quench endogenous peroxidase activity, following which incubation with a protein block for 5 min was performed to inhibit nonspecific binding. Diluted primary antibody was added and sections were incubated for 15 min. Sequential 15-minute incubations were performed with anti-mouse Ig-HRP, fluorescyl-tyramide hydrogen peroxide, and antifluorescein-HRP. Finally, the slides were incubated for 5 min with DAB/hydrogen peroxide. For CA-9, endogenous peroxidase activity was blocked using DAKO peroxidase block (Envision kit) for 5 min. Then a DAKO Protein Block (X0909) was added for a further 5 min, following which incubation with the CA-9 primary antibody diluted 1/50 in Tris-buffered saline for 30 mins was performed. A further incubation was then performed with DAKO Envision HRP Mouse polymer (K4006) for 30 min, followed by 5 min with DAB solution.

When the programme was complete, stained slides were removed from the machine and counterstained in Gills Haematoxylin (Surgipath Europe Ltd, 01500E) for 5 s. Slides were then washed in tap water, dehydrated in graded alcohols (70, 90, and 100%), cleared in xylene and mounted in DPX (Surgipath Europe Ltd, 08600E). Each staining run incorporated a control slide that had previously demonstrated positive for the antibody of interest. A negative control was also incorporated and involved the substitution of the anti-human primary antibody for an isotypic control antibody at the same protein concentration.

### Quantification of marker protein expression

Immunohistochemical staining of marker protein expression was quantified using a spectral imager developed and constructed in our Institute, as reported previously (Barber *et al*, 2003). This allowed accurate immunostain quantification, with stain intensity being expressed as optical density (OD) normalised to reference spectra. Most markers demonstrated greater expression in tumour compared with stroma, so nonspecific background staining could be minimised by applying arbitrary thresholds to the OD data. For markers exhibiting similar tumour and stromal staining, a threshold was chosen that included the staining of both tissue compartments. In addition, the number of pixels with stain intensity above the threshold was determined and represented the area of the captured image demonstrating marker expression. A standard image capture protocol was used to ensure the maximum tumour/stroma ratio was obtained for each image, which allowed comparison of stained area between captured images.

Two images were captured for each of the five biopsies taken before and after surgery, and for each captured image the stain intensity and area were determined by spectral imaging. The change in marker stain intensity was given by the difference between mean stain intensity after surgery (Ma) and mean before (Mb), whereas the change in the number of pixels demonstrating marker expression was given by Pa–Pb. Hence, a positive difference implies greater marker expression after surgery. Cyclin A is a nuclear antigen and HIF-1*α* showed mixed nuclear and cytoplasmic staining. Therefore, a labelling index (ratio of positive to negative nuclei) was calculated by counting stained cells within each captured image for HIF-1*α* and cyclin A, and the change in nuclear score for these markers was given by the difference between mean labelling index before and after surgery.

### Statistics

The Wilcoxon signed ranks test was used to determine the magnitude of overall deviation from zero of the difference between pre- and postoperative marker levels, where a significant deviation from zero would suggest an effect of surgery on marker expression. The sign test was used to calculate the significance of the deviation with respect to direction. A *P*-value of <0.05 was taken to represent significance. Spearman's rank correlation coefficient, *r*_s_, was used to determine the nonparametric correlation between variables.

## RESULTS

Samples were obtained from 30 patients undergoing rectal cancer surgery. Six patients were excluded as they had had neoadjuvant 5-FU based chemoradiation and subsequent biopsy examination revealed no residual tumour. Therefore, data for 24 untreated patients (median age 70, range 49–86; 16 male, 8 female) were available for protein analysis. The patient characteristics are shown in Table 2.

### The effect of CRC surgery on tumour protein expression

**Figure 1:**
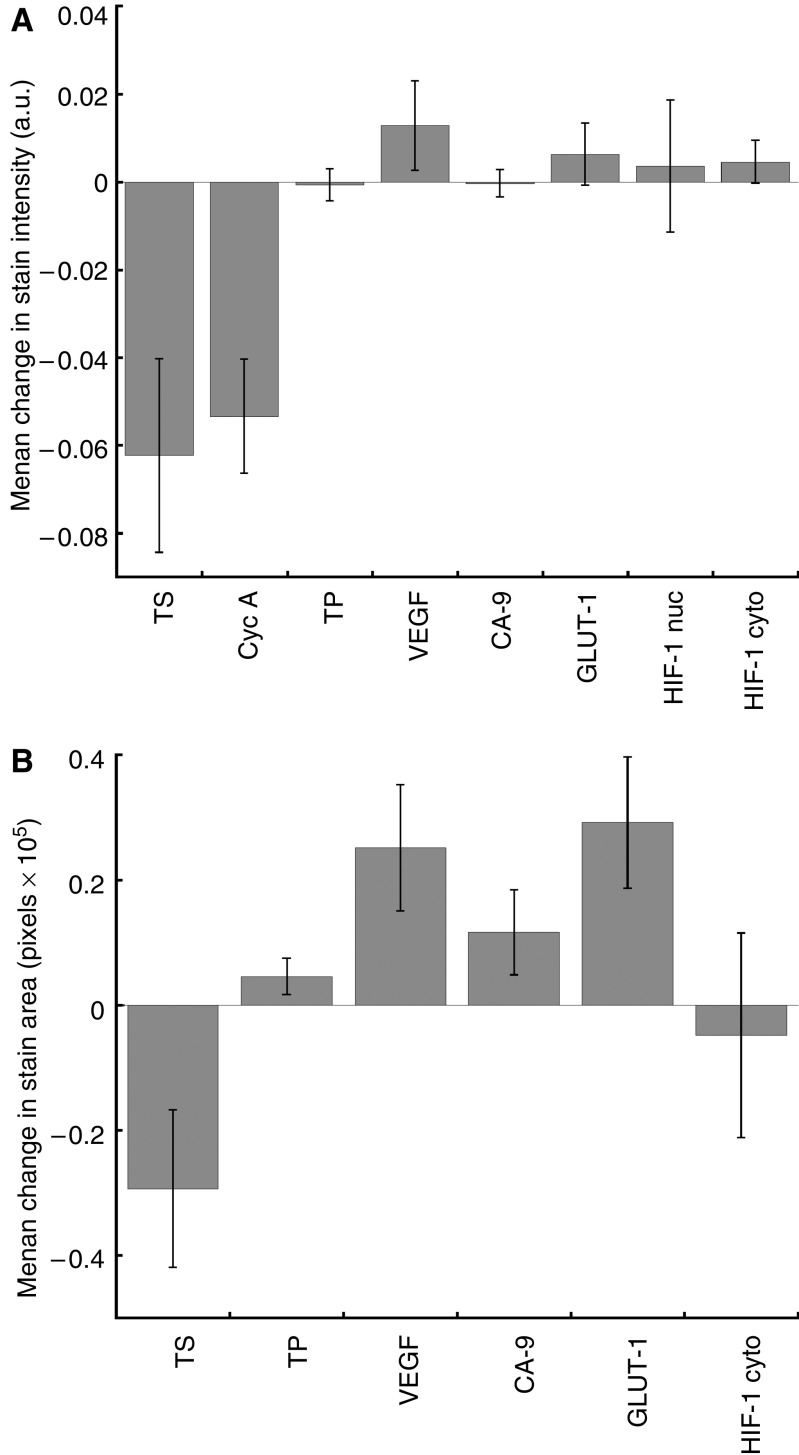
Overall changes in marker stain intensity ((**A**) arbitrary units, a.u.) and area (**B**) following surgery (error bars denote s.e.).

The overall changes in marker stain intensity and area are given in Figures 1A and B. Thymidylate synthase stain intensity in postoperative biopsies was significantly different than biopsies taken before surgery (*P*=0.02), suggesting an effect of surgery on the level of TS expression. For most patients there was a reduction in stain intensity after surgery, but this direction of change was not significant (16 patients with reduced expression postoperatively *vs* 8 patients with increased expression; *P*=0.15). Thymidylate synthase stain area also differed following surgery (*P*=0.04), with most patients showing a reduction in area (17 *vs* 7 patients; *P*=0.06). Surgery also reduced cyclin A expression (*P*=0.01), but the direction of change was not significant (18 *vs* 6 patients; *P*=0.2). The only other marker to demonstrate a change in stain intensity following surgery was the HIF-1*α* nuclear count, which increased postoperatively (magnitude: *P*=0.002; direction: *P*=0.007). GLUT-1 and VEGF stain area were altered by surgery (*P*=0.004 and 0.03 respectively), with most patients showing increased expression of GLUT-1 postoperatively (19 *vs* 5; *P*=0.007).

### The effect of CRC surgery on control protein expression

The only marker to show altered protein expression within samples of normal rectal mucosa was CA-9, which demonstrated a reduction in stain area following surgery (magnitude: *P*=0.009; direction: *P*=0.02).

### Correlations with durations of ischaemia and extirpation, and with the tumour level

**Figure 2:**
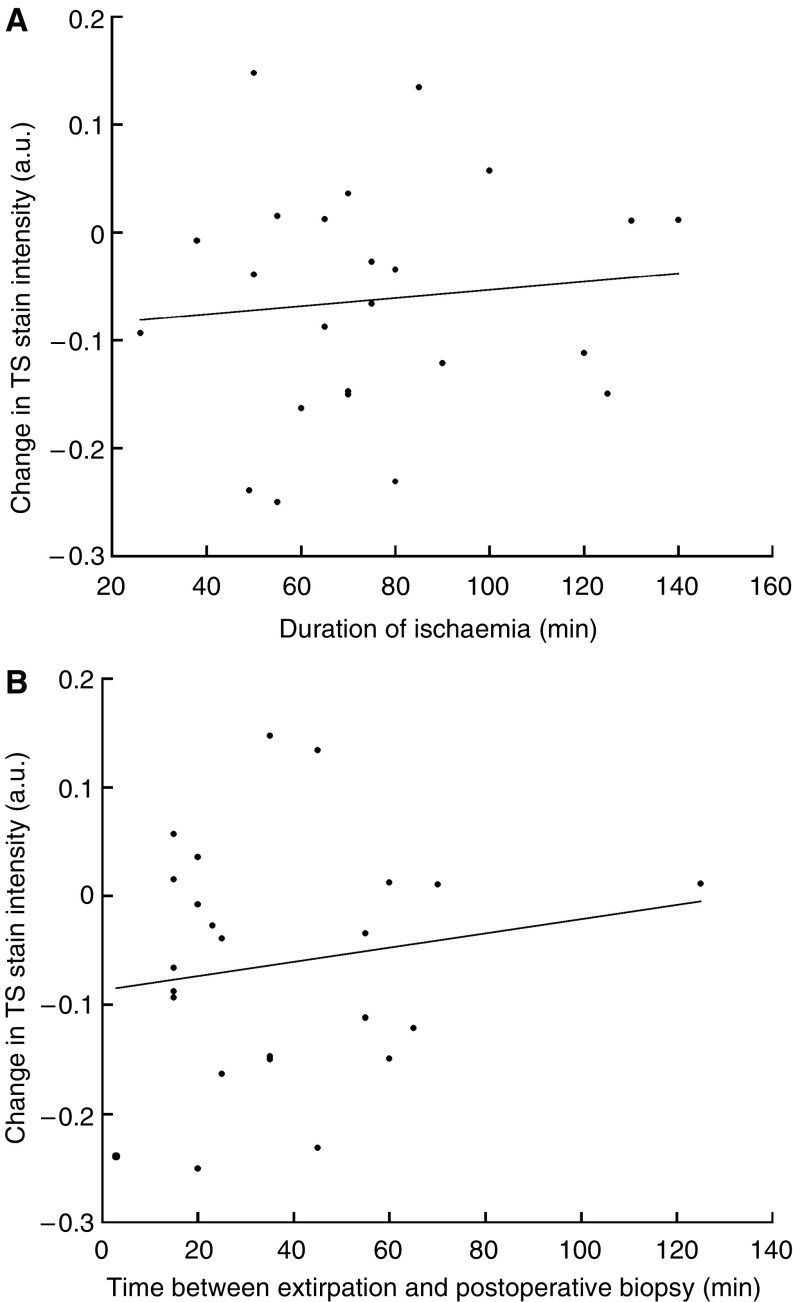
Correlation between changes in TS stain intensity (a.u.) and duration of ischaemia ((**A**) *r*_s_=0.12, *P*=0.58) and extirpation ((**B**) *r*_s_=0.06, *P*=0.77).

The median duration of tumour ischaemia was 70 min (range 26–140), while the median time between extirpation and postoperative tumour sampling was 30 min (range 3–125). There was no correlation between the change in expression and the durations of ischaemia or extirpation for any marker studied. Correlations for TS are given in Figures 2A and B. There was also no correlation between the changes in stain intensity and area and the tumour level above the anal verge. The poor correlation between changes in hypoxic marker expression and tumour level would suggest there was no direct relationship between ischaemic insult and the degree of residual vascular inflow from the middle and inferior rectal arteries following clamping of the main vascular pedicle.

### Correlations between TS expression and the markers of proliferation and hypoxia

Thymidylate synthase stain area correlated with cyclin A expression (*r*_s_=0.61, *P*=0.002), suggesting changes in TS mirrored changes in cell proliferation. Thymidylate synthase stain area also correlated with cytoplasmic HIF-1*α* and GLUT-1 stain area (*r*_s_=0.73, *P*<0.0001; *r*_s_=0.49, *P*=0.02 respectively), whereas the correlation between TS stain intensity and nuclear HIF-1*α* expression approached significance (*r*_s_=0.39, *P*=0.06).

### Impact of surgery on immunohistochemical visual grading scores

**Figure 3:**
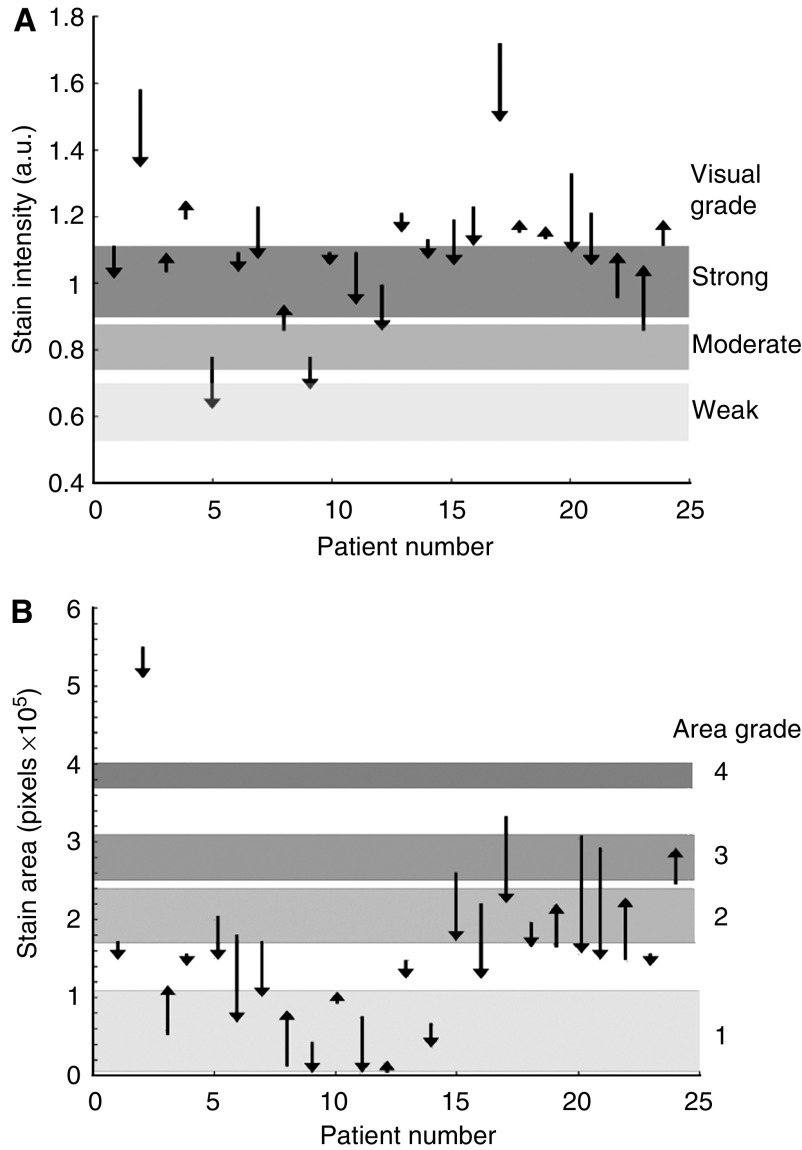
The changes in TS visual stain intensity (**A**) and area (**B**) scores occurring during CRC surgery. The length and the bearing of the arrows represent the magnitude and direction of the change in TS expression between pre- and postoperative biopsies.

Spectral imaging is a novel method of immunostain quantification, and as such is not widely available. The most common method of quantifying immunohistochemical staining patterns is by visual estimation of marker stain intensity and area. Figures 3A and B show the magnitude and direction of the changes in TS expression after surgery in relation to these manual visual grades. The limits for the visual grades are given in Table 3 and were derived by analysis of a subset of patients and reported in a previous study (Atkin *et al*, 2005). Although these limits are somewhat arbitrary, it is evident the visual intensity score is different in five patients after surgery compared with the preoperative score, whereas in six patients the visual area grades are different postoperatively. These visual scores are widely used to grade the expression of markers such as TS, and in future may be involved in guiding management decisions based on marker expression. These results suggest that surgery may alter these visual scores, and hence there may be discrepancies in marker expression in archival tumour samples compared with *in situ* levels.

## DISCUSSION

This study investigated the change in gene expression occurring during the surgical resection of primary rectal cancer. Warm ischaemia has been shown to alter the gene expression profile of a range of tissue types (Miyatake *et al*, 2004), and transcriptional changes following extirpation have been demonstrated for multiple genes within a microarray of colorectal mucosal samples beginning 20 min after excision (Huang *et al*, 2001). Extirpation was not shown to have an affect on DPD enzyme activity in CRC samples (Sadahiro *et al*, 2003); however, delays in fixation following excision have been shown to impair the analysis of the oestrogen receptor (ER) status in breast cancer patients (Von Wasielewski *et al*, 1998).

We found CRC surgery significantly altered intratumoural biomarker levels, with TS showing the greatest response. There was a trend towards a reduced expression of TS following surgery, and it was interesting to note the correlation between TS and the proliferation marker cyclin A, a finding also seen previously in an experimental model of surgically induced CRC ischaemia (Atkin *et al*, 2006). In fact, TS has been suggested to be a marker of cell proliferation, as it is induced during the G1 phase of the cell cycle and levels increase 20-fold as cells enter the S phase (Santi *et al*, 1974). Hypoxia slows the metabolic rate and is associated with lower levels of transcription and translation (Dachs and Tozer, 2000); hence, the reduction in TS levels seen after surgery may be related to the effect of tumour ischaemia on the proliferation status.

Thymidine phosphorylase (TP) is an enzyme involved in thymidine homeostasis and is involved in the activation and metabolism of the fluoropyrimidines, including 5-FU. It is also known as platelet derived endothelial cell growth factor, and has been shown to promote angiogenesis (Miyazono *et al*, 1987). High tumour TP levels have been shown to correlate with poor prognosis (van Triest *et al*, 2000), and response to 5-FU (Metzger *et al*, 1998) in CRC patients. In our study, CRC surgery did not appear to have an effect on TP expression. Previous work has demonstrated TP protein induction by hypoxia in a breast carcinoma cell line (Griffiths *et al*, 1997), but only after 16 h and there was no induction with oxygen concentrations greater than 0.3%. The same study also detected an increased TP expression after 2 h of vascular clamping in a breast tumour xenograft model. Our data, however, suggest that the degree and duration of tumour ischaemia is insufficient to alter TP levels in rectal cancer patients undergoing surgery, which is an important finding and suggests archival specimens are appropriate for TP analysis.

The absolute intratumoral oxygen tensions were not measured in this study, and so it is not possible to document the degree of ischaemia for each tumour following arterial clamping. This will vary depending on tumour level and anatomical differences in collateral blood supply. Hypoxia-related markers were measured in an attempt to determine the ischaemic insult. Hypoxia-inducible factor-1 is a transcription factor involved in oxygen homeostasis. It was noted there were changes in HIF-1*α* expression, with a significant upregulation in HIF-1*α* nuclear protein expression postoperatively. The cytoplasmic protein expression was unaltered. However, hypoxia results in translocation of the HIF-1 complex to the nucleus to allow DNA binding (Bos *et al*, 2004), and so one would expect an increased differential nuclear expression. Carbonic anhydrase-9 (CA-9) is a downstream mediator of HIF-1 activation induced by hypoxia (Wykoff *et al*, 2000). There were no changes in CA-9 expression noted in this study. However, cell line data (Lal *et al*, 2001) suggest the duration of ischaemia noted in our patients was not long enough to cause CA-9 induction. Vascular endothelial growth factor and glucose transporter-1 (GLUT-1) are also products of HIF-1 activation, and CRC surgery did have an effect on the expression of both these markers. Taken together, these data indicate a considerable tumour hypoxic insult during vascular clamping.

In an experimental model of surgically induced CRC ischaemia, TS expression correlated directly with cyclin A and inversely with CA-9 (Atkin *et al*, 2006), suggesting greater TS downregulation occurred at lower oxygen tensions secondary to a decreased cell proliferation. The direct correlation between TS and HIF-1*α* expression noted in this study therefore appears paradoxical. It may be that other microenvironmental factors, such as acidosis or alterations in the availability of glucose and other enzymatic substrates, are more active in human tumours, thereby affecting the level of TS or HIF-1*α* expression. Alternatively, it may relate to the role of HIF-1 in hypoxia-induced growth arrest (Goda *et al*, 2003), or the regulation of TS by other factors, such as p53 or E2F-1, which are induced by hypoxia and are involved in TS regulation (Ash *et al*, 1995). It was interesting to note there was no correlation for any marker between the change in expression and the durations of ischaemia and extirpation, suggesting a complex interplay between gene expression and the tumour microenvironment following vascular clamping.

The findings of this study have important implications on the timing of biomarker measurement in relation to the surgical procedure. They suggest the need for guidelines on specimen fixation following excision, and the importance of methodological standardisation of studies investigating prognostic and predictive markers, particularly if they are to be used to dictate therapeutic strategy. The results are relevant as standard surgical techniques were used and the tumour processing methods were those encountered in everyday practice. In fact, the duration of tumour ischaemia was shorter in all cases than that occurring in actual clinical practice.

Several potential sources of error were encountered. The tumour/stroma ratio varied between captured images, making it difficult to compare directly the stained area between tumour sections. However, a standard protocol for image capture was followed, ensuring each captured image contained the maximum available tumour tissue. Solid tumours show architectural heterogeneity, with varying levels of hypoxia and acidosis throughout the tissue. This introduces sampling error when measuring gene expression levels in biopsy specimens. This was minimised as far as possible by multiple sampling before and after surgery, and determining the mean marker level at each time point. Gene expression profiles of tumour biopsies are representative of the whole tumour (Perou *et al*, 2000); hence, the changes in expression seen should be applicable to the whole lesion.

The effects of tissue processing must be considered when using immunohistochemistry to measure marker levels. Tumour fixation occurs at unpredictable rates, and may take days depending on the fixative and the size of the specimen (Heyderman *et al*, 1989). Similarly, protein crosslinking during tissue fixation may alter the antigenic determinants, whereas antigen retrieval techniques may ‘unmask’ unrelated antigens (Heyderman *et al*, 1989). To reduce protein degradation during processing, large specimens should be incised to expedite fixation, or a representative tumour sample should fixed immediately and used for subsequent protein quantification. Alternatively, where delays in fixation are unavoidable, refrigeration of the specimen may minimise the loss of immunoreactivity.

## CONCLUSIONS

In patients undergoing rectal cancer surgery, significant changes in gene expression were noted between pre- and postoperative tumour biopsies. These findings have important considerations when investigating prognostic markers and markers predicting the response to adjuvant therapy. These data suggest the duration of tumour ischaemia should be minimised prior to fixation, and that subsequent studies need to standardise prognostic and predictive marker measurement in relation to the surgical procedure.

## Figures and Tables

**Table 1:** Markers studied, and conditions for immunohistochemistry

**Antibody**	**Pretreatment**	**Dilution**	**Source of antibody**
Thymidylate synthase (TS)	4 × 4 min microwave in citric acid with 10 min standing	1/300	Simon Joel, London, UK
Cyclin A	3 × 4 min microwave in citric acid with 10 min standing	1/100	#NCL-CYCLIN A, Novocastra Labs Ltd, UK
Thymidine phosphorylase (TP)	None	1/400	#MS-499-P0, NeoMarkers Inc., USA
Carbonic anhydrase-9 (CA-9)	None	1/50	Adrian Harris, Oxford, UK
Glucose transporter-1 (GLUT-1)	3 × 4 min microwave in citric acid with 10 min standing	I/200	#A3536, DAKO Corporation, USA
Hypoxia inducible factor-1*α* (HIF-1*α*)	4 × 4 min microwave in citric acid with 20 min standing	1/1000	#ab463-100, Abcam Ltd, UK
Vascular endothelial growth factor (VEGF)	3 × 4 min microwave in Tris-HCl with 10 min standing	1/100	#MS-350-P1, NeoMarkers Inc., USA

**Table 2:** Data for patients included in the analysis

**Patient**	**Age (years)**	**Sex**	**Tumour level (cm from anus)**	**Histological stage**	**Duration of ischaemia (min)**	**Duration of extirpation (min)**
1	77	F	13	T3 N0	26	15
2	72	M	10	T3 N2	49	3
3	58	M	15	T2 N0	140	125
4	64	M	10	T3 N1	65	60
5	74	F	15	T4 N2	70	35
6	60	M	10	T3 N0	75	15
7	59	M	15	T3 N2	125	60
8	80	M	5	T3 N0	70	20
9	57	M	13	T3 N1	80	55
10	71	F	15	T3 N0	38	20
11	62	M	14	T3 N0	60	25
12	77	M	12	T2 N0	120	55
13	63	M	2	T2 N1	75	23
14	86	F	13	T3 N0	50	25
15	49	F	14	T2 N0	90	65
16	82	F	3	T3 N1	65	15
17	68	F	12	T3 N0	55	20
18	71	M	10	T3 N0	55	15
19	78	M	15	T3 N0	130	70
20	76	F	5	T3 N2	80	45
21	68	M	13	T3 N0	70	35
22	64	M	15	T3 N0	85	45
23	77	M	15	T3 N0	50	35
24	55	M	14	T4 N2	100	15
						
Median	69.5		13		70	30
Range	49–86		2–15		26–140	3–125

**Table 3:** Spectral stain intensity and area limits for each visual grade (visual intensity score represents overall TS stain intensity, visual area score denotes the percentage of the section staining positively for TS)

**Grade**	**1**	**2**	**3**	**4**
Visual intensity score	Negative	Weak	Moderate	Strong
Spectral intensity range (a.u.)	0	0.53–0.66	0.75–0.88	0.91–1.11
Visual area score	<20%	20–50%	50–75%	>75%
Spectral area range (pixels × 10^5^)	0.02–1.1	1.7–2.4	2.5–3.1	3.7–4.0
